# Attenuated transforming growth factor beta signaling promotes metastasis in a model of HER2 mammary carcinogenesis

**DOI:** 10.1186/s13058-014-0425-7

**Published:** 2014-10-04

**Authors:** Sergey V Novitskiy, Elizabeth Forrester, Michael W Pickup, Agnieszka E Gorska, Anna Chytil, Mary Aakre, Dina Polosukhina, Philip Owens, Dilyara R Yusupova, Zhiguo Zhao, Fei Ye, Yu Shyr, Harold L Moses

**Affiliations:** 1 Cancer Biology Department, Vanderbilt-Ingram Cancer Center, Nashville, TN USA; 2 Department of Urologic Surgery, Vanderbilt-Ingram Cancer Center, Nashville, TN USA; 3 Department of Human Development, Peabody College, Vanderbilt University, Nashville, TN USA; 4 Division of Cancer Biostatistics, Department of Biostatistics, Vanderbilt University, Nashville, TN USA; 5 Vanderbilt University, 2220 Pierce Ave, PRB 612, Nashville, 37232 TN USA

## Abstract

**Introduction:**

Transforming growth factor beta (TGFβ) plays a major role in the regulation of tumor initiation, progression, and metastasis. It is depended on the type II TGFβ receptor (TβRII) for signaling. Previously, we have shown that deletion of TβRII in mammary epithelial of MMTV-PyMT mice results in shortened tumor latency and increased lung metastases. However, active TGFβ signaling increased the number of circulating tumor cells and metastases in MMTV-Neu mice. In the current study, we describe a newly discovered connection between attenuated TGFβ signaling and human epidermal growth factor receptor 2 (HER2) signaling in mammary tumor progression.

**Methods:**

All studies were performed on MMTV-Neu mice with and without dominant-negative TβRII (DNIIR) in mammary epithelium. Mammary tumors were analyzed by flow cytometry, immunohistochemistry, and immunofluorescence staining. The levels of secreted proteins were measured by enzyme-linked immunosorbent assay. Whole-lung mount staining was used to quantitate lung metastasis. The Cancer Genome Atlas (TCGA) datasets were used to determine the relevance of our findings to human breast cancer.

**Results:**

Attenuated TGFβ signaling led to a delay tumor onset, but increased the number of metastases in MMTVNeu/DNIIR mice. The DNIIR tumors were characterized by increased vasculogenesis, vessel leakage, and increased expression of vascular endothelial growth factor (VEGF). During DNIIR tumor progression, both the levels of CXCL1/5 and the number of CD11b+Gr1+ cells and T cells decreased. Analysis of TCGA datasets demonstrated a significant negative correlation between *TGFBR2* and *VEGF* genes expression. Higher *VEGFA* expression correlated with shorter distant metastasis-free survival only in HER2+ patients with no differences in HER2-, estrogen receptor +/- or progesterone receptor +/- breast cancer patients.

**Conclusion:**

Our studies provide insights into a novel mechanism by which epithelial TGFβ signaling modulates the tumor microenvironment, and by which it is involved in lung metastasis in HER2+ breast cancer patients. The effects of pharmacological targeting of the TGFβ pathway *in vivo* during tumor progression remain controversial. The targeting of TGFβ signaling should be a viable option, but because VEGF has a protumorigenic effect on HER2+ tumors, the targeting of this protein could be considered when it is associated with attenuated TGFβ signaling.

**Electronic supplementary material:**

The online version of this article (doi:10.1186/s13058-014-0425-7) contains supplementary material, which is available to authorized users.

## Introduction

Transforming growth factor-beta (TGFβ) is a homodimeric polypeptide, which includes three isoforms: TGFβ1, TGFβ2 and TGFβ3. Secreted TGFβ binds to TGFβ receptor II (TβRII) and forms a heterodimeric complex with TGFβ receptor I (TβRI). The activated TβRI phosphorylates intracellular Smad2 and Smad3 (canonical TGFβ pathway). Simultaneously, phosphorylation of TβRII activates PI3K, MAP3k1, PP2A, RHOA and others (non-canonical pathway) [1]. TGFβ plays a major role in the regulation of tumor initiation, progression, and metastasis, which requires TβRII for signaling [1].

It has been published that decreased expression or loss of TβRII correlates with an increased risk of developing invasive breast cancer [2]. Contrary to this fact, in mouse models of cancer, the inhibition of TGFβ signaling with the expression of dominant-negative TβRII (DNRII) or deletion of TβRII increases cellular proliferation without initiating tumor development [3],[4]. Therefore, the assumption is that attenuated TGFβ signaling alone is insufficient for transformation. In our previous research article it was indicated that deletion of TβRII in mammary epithelial of mouse mammary tumor virus (MMTV)-polyoma middle T antigen (PyMT) mice results in shortened tumor latency and a five-fold increase in lung metastases compared to MMTV-PyMT tumors with intact TGFβ signaling [5],[6]. The mechanisms behind this phenotypic difference are correlated with the increased expression of CXCL1, CXCL5 and CCL20 [7],[8]. Abrogated TGFβ signaling in carcinoma cells can indirectly promote progression of MMTV-PyMT tumor and metastasis by polarization T cells to Th17 cells via accumulation of CD11b^+^Gr1^+^ cells [9]. Additionally, epithelial TGFβ signaling regulates fibroblast recruitment and activation. Our recent article confirmed the fact that fibroblast-stimulated carcinoma cells utilize TGFβ signaling to drive single-cell migration, but migrate collectively in the absence of TGFβ signaling, which promotes mammary tumor invasion [10].

Mammary tumorigenesis has been examined through the use of numerous transgenic mouse models with wide utilization of the MMTV promoter/enhancer to drive expression in mammary epithelium. Overexpression of ErbB2 (Neu, human epidermal growth factor 2 (HER2)) or a constitutively active version of this receptor in the mammary epithelium leads to the development of metastatic mammary tumors [11]-[13]. Concurrently, overactivation of the ErbB2 pathway correlates with poor clinical prognosis in breast cancer patients [14]. Using Neu-induced mammary tumor models with increased activity of TGFβ signaling (MMTV/ALK5 and MMTV/TGFβ1), it was possible to induce that active TGFβ signaling accelerates metastasis and the number of circulating tumor cells [15]-[17]. The loss-of-function experiments through the expression of soluble betaglycan or a DNIIR has been reported to suppress metastasis in Neu-induced mammary tumors [16],[18].

Based on these data we decided to examine the connection between TGFβ and Neu signaling in mammary tumor progression using MMTV-Neu and MMTV-Neu^activated^ induced tumorigenesis [13]. The transgenic strains in conjunction with mice expressing DNIIR were used in the mammary epithelium to investigate the effect of attenuated TGFβ signaling on tumorigenesis and metastasis. We found that attenuation of TGFβ signaling with DNIIR prolonged tumor latency and dramatically enhanced pulmonary metastasis. The mechanism was different from that reported for the MMTV-PyMT model with conditional deletion of TβRII. Increased chemokine secretion through the knockout of carcinoma cells with resultant influx of CD11b^+^Gr1^+^ myeloid cells increased metastasis [9]. In the MMTV-Neu model with DNIIR, there was no difference in chemokine secretion increase by the carcinoma cells and no increase in immature myeloid cell infiltration. Instead, there was reported increased secretion of vascular endothelial growth factor (VEGF), diminished pericyte coverage of vessels, and increased vessel leakiness and vasculogenesis. These symptoms likely act as the mechanism for the increased number of metastases. Lastly, analysis of human breast cancer transcriptome databases demonstrated a significant correlation between decreased *TGFBR2* and increased *VEGFA* gene expression similar to what was observed in the mouse models. Higher *VEGFA* gene expression was correlated with poor survival only in HER2-positive (HER2+) patients.

## Methods

### Mice and cell lines

All studies were performed on 202Mul and NK1Mul mice. To generate the mice with DNIIR-dominant-negative TβRII (202Mul/DN and NK1Mul/DN) 202Mul or NK1Mul mice ordered from Jackson Laboratory (Bar Harbor, ME, USA) and mice with expressed dominant-negative TβRII were crossed [19]. The mice are proven to be on pure FVB background. The studies were approved by IACUC at Vanderbilt University Medical Center, Nashville, TN, USA.

The 202Mul and 202Mul/DN carcinoma cell lines were derived from primary tumors of 202Mul and 202Mul/DN mice, established and cultured in DMEM/F12 with 5% adult bovine serum. These carcinoma cells were implanted into the mammary fat pad of the #4 mammary gland via collagen plugs (CP) (5 × 10^5^ cells/plug). CP were prepared by suspending carcinoma cells (5 × 10^5^/plug) in collagen solution (50 mkl/plug), followed by pipette of this mixture (50 mkl) to 12-well dishes and incubation at 37°C for 45 minutes to solidify the gel. Then CP were overlaid with medium and incubated for an additional 4 to 18 hours at 37°C. The collagen mixture contained rat-tail collagen type I (BR Biosciences, San Jose, CA, USA), 10 × Earle's Balanced Salt Solution (EBSS, Gibco), NaHCO3, 1 M NaOH, and sterile ddH_2_O. The size of tumors was determined by direct measurement of tumor dimensions at 2 to 3 day intervals using calipers.

### Flow cytometry analysis

Single-cell suspensions were made from the spleens of tumor-bearing mice [20],[21], and tumor tissues [22]. Excised tumors were chopped into small pieces, incubated in DMEM (Gibco, Life Technologies, Grand Island, NY, USA) with no serum, 1 mg/mL collagenase I (Sigma, St. Louis, MO, USA), and 1 mg/mL Dispase II (Roche) for 2 hours at 37°C, and then passed through a cell strainer. Total cell numbers were counted, and CD45^+^ cell populations that represented tumor-infiltrating host immune cells were analyzed by flow cytometry. After treatment with FcR Blocking Reagent (Miltenyi Biotec Inc., Auburn, CA, USA), tumor single-cell suspensions (10^6^ cells/mL) were labeled using fluorescein-conjugated antibodies (Abs) (Biolegend, eBiosciense, BD, all from San Diego, CA, USA) for 20 minutes on ice. Data acquisition was performed on a LSRII flow cytometer (BD Immunocytometry Systems, Franklin Lakes, NJ, USA), and the data were then analyzed with FlowJo software. Nonviable cells were excluded using 4',6-diamidino-2-phenylindole (DAPI). Antigen negativity was defined as having the same fluorescent intensity as the isotype control.

### ELISA

Cytokine levels in conditional media and tissue lysates were measured using the mouse CXCL1, CXCL5, MCP-1, VEGF, and IL-6 ELISA Duo kits (R&D Systems, Minneapolis, MN, USA) following the manufacturer's protocol.

### Histology, IHC, and IF staining

Tissues were embedded directly in an optimal cutting temperature compound without fixation or placement in 10% formalin overnight, and then embedded in paraffin and sectioned at 5 μm. Sections were de-waxed in xylene and rehydrated in successive ethanol baths. For immunohistochemistry (IHC), the MOM kit was used (Vector). H&E and CD34 staining were performed in Translational Pathology Shared Resources (Vanderbilt University, Nashville, TN, USA). For immunofluorescence (IF) staining, primary and secondary antibodies were diluted in 12% BSA, and then mounted in DAPI that contained a SlowFade medium (Invitrogen). Antibodies used for staining were NG2 (1:200; Abcam), CD31 (1:200; BD Biosciences), 5-bromo-2’-deoxyuridine (BrdU) (BD Biosciences). Quantification of staining was performed using ImageJ software (National Institutes of Health, Bethesda, MD, USA) in accordance with the recommended guidelines. H&E and IHC sections were photographed using the OLYMPUS BX41 microscope and OLYMPUS DP2-BSW software. Slides for H&E and CD34 staining of lungs were scanned using the Leica SCN400 slide scanner with 20 × objective. Slides were photographed using a ZEISS Axioplan 2 microscope, and then numbered using MetaMorph software.

### Whole-lung mounting

Mice were sacrificed by anesthetic overdose. Lungs were processed as described in the previously published article [23]. The tumor nodules in lungs were then counted.

### Cytokine antibody array

Cells (10^6^) were plated on a 6-wells plate in 3 mL of DMEM/F12 with 5% of adult bovine serum. Conditional medium was collected after 18 hours, and secreted proteins were screened using the RayBio Mouse Cytokine Antibody Array C Series 1000 (RayBiotech Inc., Norcross, GA, USA) according to the manufacturer's instructions.

### Western blot analysis

Cells or tissue were lysed in radioimmunoprecipitation assay buffer containing protease inhibitors cocktail (Roche Diagnostics, Indianapolis, IN, USA). Total protein concentrations were quantified with the Pierce BCA Protein Assay Kit (Pierce Biotechnology, Rockford, IL, USA). Equal amounts of protein (30 to 60 μg/well) were resolved in NuPAGE Novex 4 to 12% Bis-Tris polyacrylamide gel in the presence of 1 × MES buffer (2-(*N*-morpholino)ethanesulfonic acid; Invitrogen) and transferred to a polyvinylidene fluoride membrane Immobilon-FL (Millipore Bioscience Research Reagents, Temecula, CA, USA). Anti-Akt (Cell Signaling, 9272), ph-Akt (Cell signaling, 4060), actin (Sigma, A2066) and secondary anti-Rabbit (Thermo Scientificm 31462) were used at 1:1,000, 1:1,000, 1:2,000 and 1:5,000 dilutions, respectively. After treatment with appropriate peroxidase-conjugated secondary antibody, the bands were visualized with an enhanced chemiluminescence method [24]. The intensity of protein bands was quantified by a densitometer using ImageJ 1.45's software (National Institutes of Health).

### Proliferation assays

For *in vivo* experiments BrdU incorporation was used by injecting 100 μL of 1 mg/mL BrdU 2 hours prior to performing euthanasia of animals. For *in vitro* experiments ^3^H-thymidine incorporation was performed for 2 hours prior to conducting measurement with a scintillation counter, whereby mean cpm were normalized to untreated cells. Cells were plated in 24-well culture dishes at 4 × 10^4^ per well. TGFβ1 (R&D Systems, Minneapolis, MN, USA) treatment was performed in normal serum-containing media for 24 hours.

### Statistical analysis

Data were presented as mean ± standard error of the mean (SEM). Multiple comparisons between the treatment groups and the control untreated group were performed using one-way analysis of variance (ANOVA) followed by Dunnett's procedure for multiplicity adjustment. Two-group comparisons were performed using the two-sample *t*-test. Among the 1,056 human breast tumor tissue samples from The Cancer Genome Atlas Breast Cancer (TCGA BRCA) depository, 531 samples have both gene expression and clinical data available and were therefore used for the following analysis. Samples with low versus high TβRII expression were compared to their CXCL1, CXCL5, MCP-1, IL-6, and VEGF expression levels usingthe Wilcoxon rank-sum test for all patients as well as stratified by estrogen receptor (ER), progesterone receptor (PR), and HER2 status separately. The findings were validated using six independent GEO datasets (4992, 6532, 2990, 12093, 3494, and 10886). However, HER2 status was not available in these GEO datasets. Breast cancer subtype classifiers were available in the literature. In this study patient subtype was predicted using the PAM50 classifier (*R* package, genefu 1.0.9 [25]-[27]). The association between distant metastasis-free survival (DMFS) and VEGF was analyzed using publicly available databases (GEO, EGA, and TCGA) for breast, ovarian, and human lung tumors from [28]. All tests are two-sided and significant at the 5% level. All statistical analysis for human breast cancer data was performed in R 3.0.2 [29].

## Results

### Attenuated TGFβ signaling increases tumor latency and metastasis in neu-induced mammary tumorigenesis

**Figure 1:**
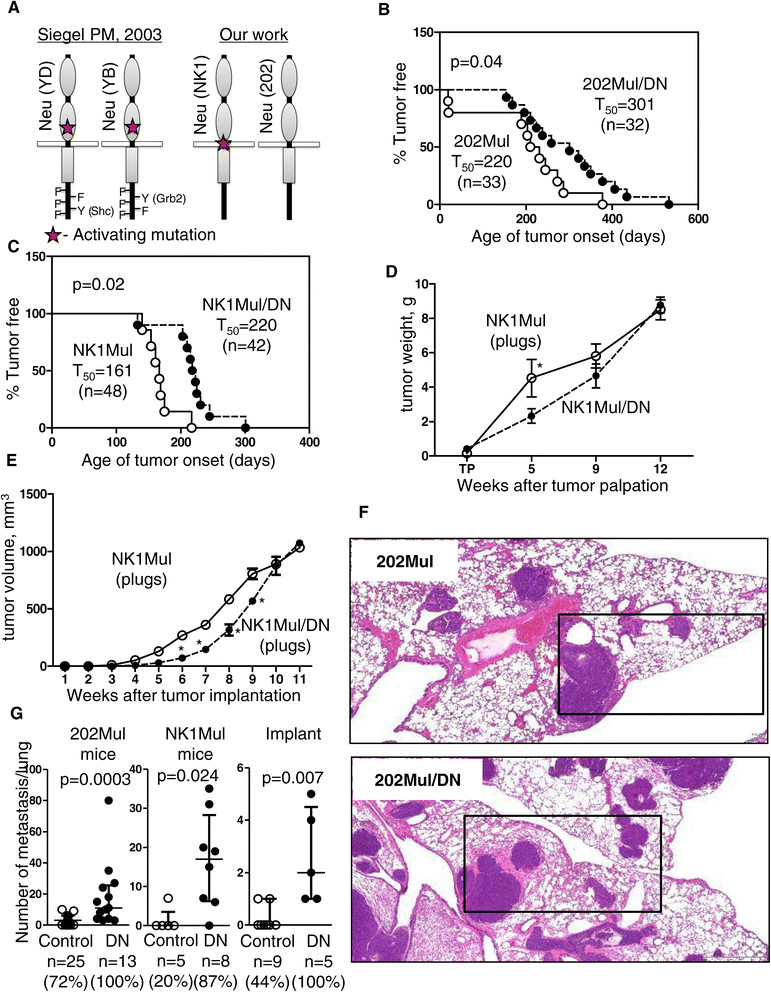
**Tumor latency and metastasis in mouse mammary tumor virus (MMTV)-Neu mice with dominant-negative (DN) transforming growth factor β (TGFβ) receptor II (DNIIR).** **(A)** Differences in two MMTV-Neu tumor models between a previously published model [16] and the models used in the current study. **(B)** Mammary tumor latency in 202Mul versus 202Mul/DN mice and **(C)** NK1Mul versus MK1Mul/DN mice. Age of onset is the time that a palpable mammary tumor first appears. *T*_*50*_ denotes the age at which 50% of mice first possess a tumor, and *n* is the number of mice examined. **(D)** Weight of tumor tissue in NK1Mul and NK1Mul/DN mice at different time points after tumor palpation. The total weight of tumors from all 10 mammary glands is indicated. **(E)** Tumor volume measured by caliper every 3 days after implantation into mammary fat pad via collagen plugs containing MMTV-Neu or MMTV-Neu/DNIIR carcinoma cell lines from NK1Mul or NK1Mul/DN mice, respectively. Five mice per group were used. **(F)** Representative H&E sections illustrated metastasis in lungs of 202Mul and 202Mul/DN mice. Black box indicates area selected to represent CD34 IHC staining in Additional file 1: Figure S1. **(G)** Number of metastatic foci in lungs by using different tumor models: 202Mul mice, Nk1Mul mice and orthotopic implantation of carcinoma cells. Data shown on a scatter plot with median and interquartile range; *n* is the number of mice examined; percentage indicates number of mice with metastasis; non-parametric Mann-Whitney test.

In our study, we used two MMTV-Neu-induced genetically engineered mouse (GEM) models of mammary cancer. Mice expressing inactivated ErbB2 (202Mul) [11] and mice expressing constitutively activated ErbB2 (NK1Mul) [13] were crossed with mice expressing a DNIIR [30]. Mice with intact TGFβ signaling in the mammary gland were the control group (202Mul, NK1Mul), and mice with modified TGFβ signaling comprised the experimental group (202Mul/DN, NK1Mul/DN). Our tumor models were slightly different compared to the models published several years ago by other investigators (Figure 1A) [16], where the authors used the MMTV-Neu mouse model with Y1144 (YB) and Y1227 (YD) mutation in ErbB2 to activate specific HER2 signaling pathway - Shc or Grb2, respectively [31].

Median tumor latency for 202Mul mice was 220 days (Figure 1B) and for activated Neu it was 161 days (Figure 1C). The addition of a DNIIR increased tumor latency to 301 days, *P* = 0.04, for 202Mul/DN mice and to 220 days, *P* = 0.02, for NK1Mul/DN mice. Morphology of tumor tissue was analyzed at 5, 9, and 12 weeks after tumor palpation. No significant difference was observed in tumor weight at the last time point (12 weeks) between control and experimental groups (Figure 1D). Nonetheless, during initial stages of the experiment (5 weeks), tumors appeared to grow slower in DNIIR mice in comparison with the control mice.

In parallel with spontaneous tumorigenesis, we established mammary carcinoma cell lines and implanted them into mammary fat pads via CP. We observed similar kinetics to our spontaneous model where tumors expressing DNIIR grew slowly in the beginning of experiment, but achieved the same size as those without DNIIR expression by the time of sacrifice (Figure 1E).

To analyze lungs for metastasis, we sacrificed mice 12 weeks after conducting tumor palpation or implantation of carcinoma cells to the mammary fat pad. In all models, we observed a significant increase in the number of lung metastasis: 5.5 times higher in 202Mul mice (*P* <0.001), almost 12 times higher in mice with activated Neu (*P* = 0.02) and 6.7 times higher in mice with orthotopic implantation of mammary carcinoma cells (*P* = 0.01) (Figure 1F, 1G). IHC staining for CD34 showed that most metastases were extravascular with increased vasculogenesis in mice with DNIIR (Additional file 1: Figure S1).

### Attenuated TGFβ signaling in the mammary gland increases tumor angiogenesis

**Figure 2:**
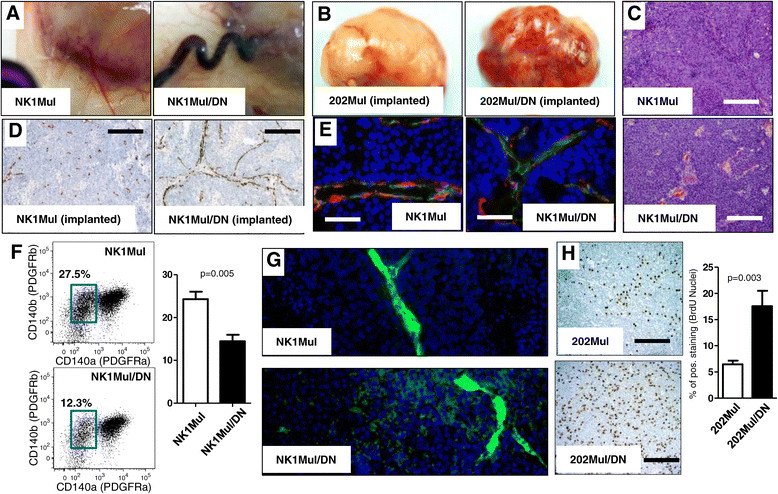
**Increased angiogenesis and proliferation in mammary carcinomas of mouse mammary tumor virus (MMTV)-Neu mice with dominant-negative (DN) transforming growth factor β (TGFβ) receptor II (DNIIR).** **(A)** Autopsy of mice with spontaneous tumor formation of the mammary gland, which indicates increased tumor vasculature in mice with DNIIR expression. **(B)** Isolated tumor tissue on 12^th^ week after implanted carcinoma cells via collagen plugs to the mammary fat pad. **(C)** H&E staining indicated morphological changes in control (NK1Mul) and experimental mice (NK1Mul/DN). **(D)** Immunohistochemistry (IHC) staining of frozen sections for endothelial cell marker CD31. Staining was performed with hematoxylin (blue) to counterstain all nuclei. **(E)** Immunofluoresence staining of frozen sections for the endothelial cells CD31 (green), pericyte marker NG2 (red) and nuclei by 4',6-diamidino-2-phenylindole (DAPI) (blue). **(F)** Representative fluorescence-activated cell sorting plots and quantitative analysis of pericyte percentage (PDGFRβ^+^PDGFRα^−^) in tumor tissue. Cells were gated as non-immune, non-epithelial (CD45^−^CD326^−^). **(G)** Formalin-fixed paraffin-embedded sections of fluorescein isothiocyanate (FITC-)dextran (green)-perfused tumor tissue for indication of vessel leakage. DAPI (blue) was used to counterstain all nuclei. **(H)** IHC for 5-bromo-2'-deoxyuridine (BrdU) on primary tumors, which indicate newly synthesized DNA in proliferating cells. Quantification of IHC (right) BrdU-positive cells indicates 2.5 times more positive cells in mice with DNIIR than control tissue section. (Scale bars: C, D, E, J, K, 500 μM; F, 1,000 μM).

Tumor latency was the only significant difference observed in the two different Neu-induced tumor models NK1Mul versus 202Mul and NK1Mul/DN versus 202Mul/DN (Figure 1B, C). Upon sacrifice, we found that vessels coming into and out of the tumor tissue are significantly larger in mice with DNIIR than in MMTV-Neu mice (Figure 2A). In orthotopically implanted CP with carcinoma cells, we found visually more abundant vasculature in mice with NK1Mul/DN tumors versus NK1Mul (Figure 2B). Examination of the histopathology of the primary tumor showed typical adenocarcinoma common to the MMTV-Neu models for both type of mice (Figure 2C). NK1Mul/DN tumors exhibited multiple zones of necrosis.

Also, we analyzed vessel structure by IHC staining for CD31, as a marker of endothelial cells, (Figure 2D) and found that vessels in DNIIR mice are larger in diameter with necrosis between them. Number of large vessels was dramatically increased in mice with DNIIR (Additional file 1: Figure S2). Similar to the primary tumor, lung metastases of DNIIR tumors have also increased vessel presence as observed in CD34 staining (Additional file 1: Figure S1). Visual increase in angiogenesis in mice with DNIIR (Figure 2B), encouraged us to analyze pericyte coverage of vessels in tumor tissue. We found fewer vessels wrapped by pericytes in DNIIR mice (Figure 2E). To confirm this finding, we performed flow cytometry analysis of pericyte number in tumor tissue (Figure 2F, Additional file 1: Figure S2). Single-cell suspensions of tumor tissue were gated for non-immune (CD45^−^), non-epithelial (Ep-CAM^−^) cells and detected as PDGFRβ^+^PDGFRα. The number of these cells was decreased two-fold in NK1Mul/DN tumors versus NK1Mul tumors (*P* = 0.005). Based on increased angiogenesis, larger vessels, and deceased number of pericytes, we hypothesized that vessels in DNIIR mice would be leakier. To test this hypothesis, we performed intravenous injection of fluorescein isothiocyanate (FITC)-dextran and analyzed tumor tissue after tumor perfusion. IF imaging clearly indicated that Neu-induced tumors with DNIIR increased vessel leakage (Figure 2G). Thus, we propose that this is a potential factor for multiplication of lung metastases in DNIIR mice.

As we observed earlier, tumor weight and size were the same on the day of mouse sacrifice (Figure 1). However, we found that the proliferation rate on BrdU staining increased in mice with DNIIR (Figure 2H). DNIIR tumors grew slower after delayed tumor latency, but then presented increased tumor proliferation, potentially due to increased angiogenesis. As a result, DNIIR tumors caught up in size with MMTV-Neu tumors.

### Attenuation of TGFβ signaling reduces chemokine, but not VEGF expression, in the tumor microenvironment

**Figure 3:**
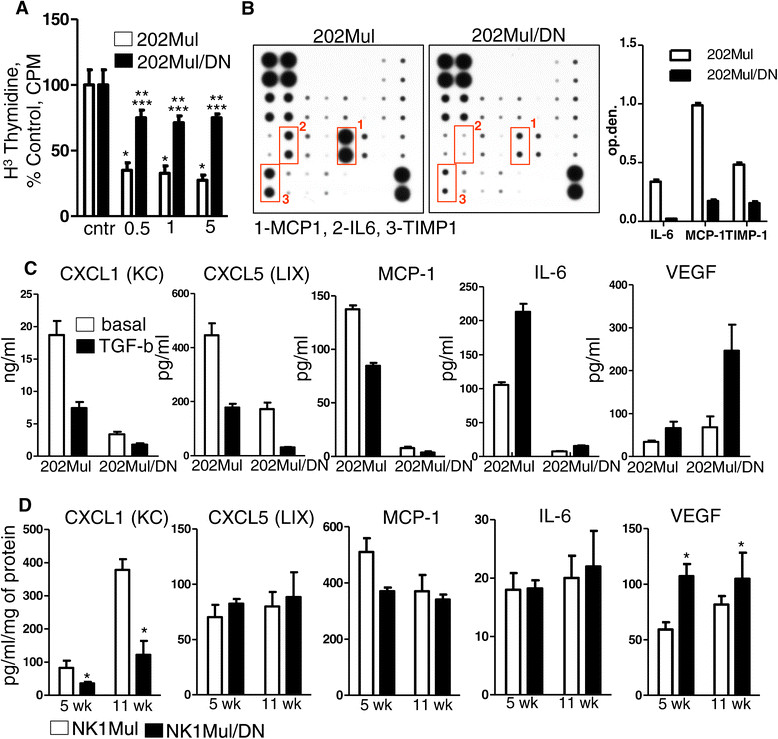
**Cytokine and chemokine profile of Erbb2+ mammary carcinoma cells with intact and modified transforming growth factor β (TGFβ) signaling.** **(A)** Thymidine incorporation assay. Epithelial cells were treated with 0.5, 1 and 5 ng/mL TGFβ and analyzed for changes in cell proliferation by ^3^(H) thymidine incorporation. Statistical significance was determined by *P*-values <0.05; *202Mul treated versus 202Mul untreated, **202Mul/DN treated versus 202Mul/DN untreated, ***202Mul/DN treated versus 202Mul treated. **(B)** Representative mouse cytokine array using tumor explant supernatants [45] from 202Mul and 202Mul/DN cell lines (left) quantification of the mouse cytokine array data for IL-6, monocyte chemotactic protein 1 (MCP1) and tissue inhibitor of metalloproteinase 1 (TIMP1) (right). **(C)** ELISA of conditional medium from 202Mul and 202Mul/DN cell lines untreated and treated for 24 hours with TGFβ 1 ng/mL. All changes in cytokine and chemokine secretion were significant (*P* <0.05) in 202Mul versus 202Mul/DN cell lines and treated versus untreated with TGFβ. **(D)** ELISA of protein extracts from tumor tissue homogenates isolated from NK1Mul and NK1Mul/DN mice on 5 and 11 weeks after tumor palpation. Five mice per group were used excluding vascular endothelial growth factor (VEGF) measurement, where eight mice were used; *P <0.05. Data correspond to the mean ± standard error of the mean.

A classically defined role of TGFβ is the induction of cell-cycle arrest. To test the ability of DNIIR expression to attenuate this phenotype, we incubated established mammary carcinoma cell lines from 202Mul and 202Mul/DN mice with TGFβ for 24 hours at different concentrations. Starting from 0.5 ng/mL of TGFβ, we observed inhibition of cellular proliferation in the presence of intact TGFβ signaling. In 202Mul/DN cells, TGFβ had an inhibitory effect compared with untreated cells, but a smaller magnitude of change in comparison with 202Mul cells (Figure 3A).

In PyMT-induced mammary tumorigenesis, deletion of TβRII was associated with a strong increase in secretion of CXCL1 and CXCL5 chemokines from mammary carcinoma cells [7]-[9] and significantly correlated with increased tumor progression and metastasis formation. We performed a protein array on tumor explant supernatants and found a significant decrease of monocyte chemotactic protein (1 MCP1) (CCL2), IL6, and tissue inhibitor of metalloproteinase (1 TIMP1) expression in cells expressing DNIIR (Figure 2B). To specify the cellular origin of chemokine changes, we used conditioned medium from cultured 202Mul and 202Mul/DN cells with/without TGFβ stimulation (Figure 3C). As in the tumor tissue, TGFβ upregulated IL-6 and, VEGF, and downregulated MCP1, CXCL1, and CXCL5 in cells with and without DNIIR expression. Surprisingly, in cells with attenuated TGFβ signaling, we found dramatically decreased levels of CXCL1 and CXCL5, which were additionally downregulated by TGFβ. DNIIR expression had a significant effect on VEGF secretion. Basal level of this cytokine was increased in 202Mul/DN cells compared to 202Mul cells and was strongly upregulated by TGFβ, which correlated with the increased angiogenesis in mice with DNIIR (Figure 2A). To determine to what extent the levels of these chemokines change during tumor progression, we analyzed tumor tissue homogenates at 5 and 11 weeks after tumor palpation in NK1Mul and NK1Mul/DN mice (Figure 3D). The same effect was observed with CXCL1 and, more importantly, with VEGF. Comparison analysis of chemokines and VEGF secretion in MMTV-PyMT cells versus MMTV-Neu cells showed the same tendency, namely downregulation of CXCL5 and upregulation of VEGF in cells with DNIIR versus deletion of this receptor (Additional file 1: Figure S3).

### Attenuated TGFβ signaling in the MMTV-Neu tumor model decreases T cells and CD11b+Gr1+ cells in tumor tissue

In our previous work on MMTV-PyMT tumors, we found that abrogation of TGFβ signaling in mammary epithelium increased chemokine production and the number of CD11b^+^Gr1^+^ cells in tumor tissue, which correlated with the increased metastasis [7],[8]. Attenuated TGFβ signaling in Neu-induced tumorigenesis leads to different cellular responses, namely significant downregulation of CXCL1 and CXCL5. We analyzed tumor tissue and spleen from mice at 12 weeks after tumor palpation and found no differences in the number of T cells (CD3) and B cells (CD19) in the spleen (Additional file 1: Figure S4A). When we analyzed subsets of T cells (CD4 and CD8) we detected decreased number of T helpers (CD4) as well as an altered ratio of CD4+ to CD8+ cells (Additional file 1: Figure S4B). Expected differences in CD11b^+^Gr1^+^ cells were not found in the spleen (Additional file 1: Figure S4C).

In our analysis of tumor tissue, the total number of immune cells (CD45^+^) did not alter (Additional file 1: Figure S2D). When we analyzed fluorescence-activated cell sorting (FACS) plots for CD11b and Gr1 staining we found no differences in the number of macrophages (CD11b^+^Gr1^-^F4/80^+^), but detected a decreased number of CD11b^+^Gr1^+^ cells (Additional file 1: Figure S4E). This result was predicted due to downregulation of CXCL1 and CXCL5. Analysis of lymphocytes showed a decreased number of T cells (CD3) in NK1Mul/DN mice compared with the mice with intact TGFβ signaling. Because of the decreased number of CD11b^+^Gr1^+^ cells that can successfully inhibit T cell proliferation, we propose that the mechanism driving the decrease in T cells is not related to CD11b^+^Gr1^+^ function and may be linked to the increase of VEGF in tumor tissue [32].

### Attenuated TGFβ signaling in HER2+ tumors leads to increased pAKT

**Figure 4:**
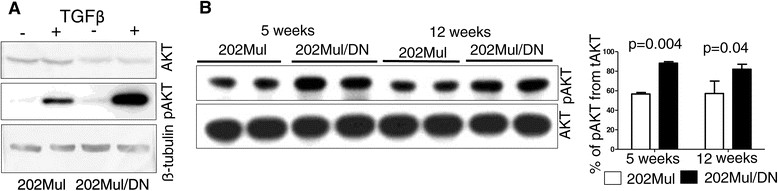
**Western blot analysis of carcinoma cell lines and tumor tissues.** **(A)** Western blot analysis of AKT and pAKT in 202Mul and 202Mul/DN established mammary carcinoma cell lines incubated with transforming growth factor β (TGFβ) 1 ng/mL for 24 hours. **(B)** Western blot and quantitative analysis of AKT and pAKT in tumor tissue lysates at 5 and 12 weeks after tumor palpation. Open bars, 202Mul mice; closed bars, 202Mul/DN mice. Protein lysates for **(A)** and **(B)** were prepared through homogenization in Complete-M lysis buffer (Roche) and quantified by Bradford DC assay (Biorad). AKT and pAKT were detected using rabbit monoclonal antobody (Cell Signaling).

There are numerous molecular mechanisms for the interaction between TGFβ and HER signaling during tumor growth, which were summarized in a recently published review [33]. We performed analysis for known components linking these two pathways. We then cultured carcinoma cells with TGFβ1 (1 ng/mL) overnight and found increased phosphorylated AKT in cells with DNIIR compared to those without DNIIR (Figure 4A, Additional file 1: Figure S5). To determine differences in pAKT in tumor tissue, we prepared tissue homogenates at 5 and 12 weeks after tumor palpation. Total AKT did not change, but we found increased pAKT in 202Mul/DN tumors compared to 202Mul tumors (Figure 4B). Quantitative data revealed significant differences at two time points, at 5 and 12 weeks of tumor progression in 202Mul/DN mice versus 202Mul mice. Additional western blot analysis did not exhibit any differences in p53, PI3K, Rac1, Shc, ERK, MAPK or p38 expression with or without DNIIR expression (data not shown).

### Human data sets reflect similar findings with the mouse models

**Figure 5:**
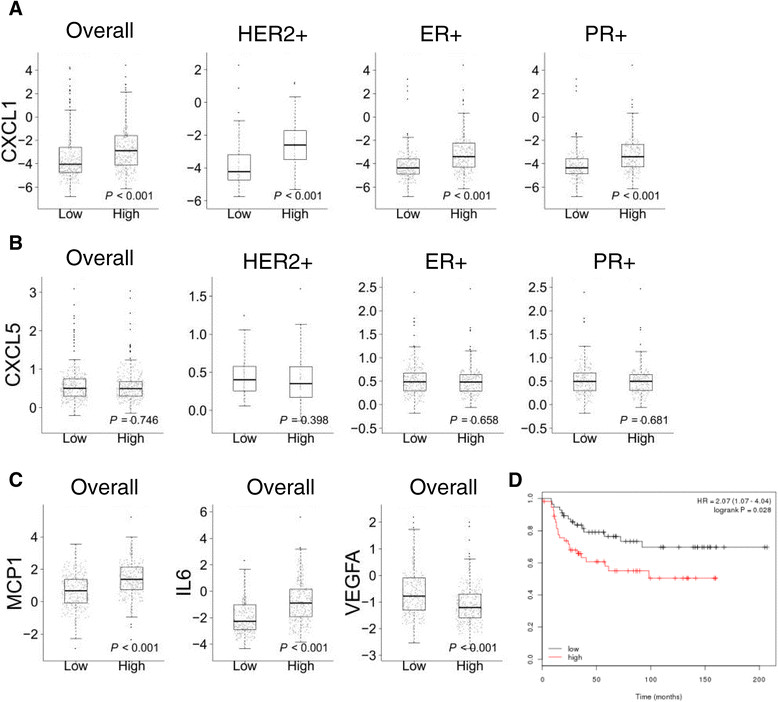
**Analysis of the relationship of transforming growth factor β receptor II (TβRII)** **, vascular endothelial growth factor (VEGF)A and chemokine expression in human datasets.** Expression of CXCL1 **(A)**, CXCL5 **(B)**, monocyte chemotactic protein (MCP)-1, IL-6 and VEGF **(C)** in human breast tumor tissues with low and high expression of TβRII using TCGA BRCA data. **(D)** In human human epidermal growth factor receptor 2-positive (HER2+) cancer patients (n = 207), the expression of *VEGFA* correlated with reduced distant metastasis-free survival (DMFS) in datasets from [28] including information from 3,455 patients [28]. All samples were plotted with medium splitting for *VEGFA* expression. No significant differences in DMFS were observed in association with *VEGFA* expression in other subtypes of breast cancer. ER+, estrogen receptor-positive; PR+, progesterone receptor-positive.

To determine the relevance of our findings to human breast cancer, we analyzed microarray profiles of human breast cancer tissues with well-documented clinical data related to PR, ER and HER2 status and time of relapse detection over a 10-year period. In our mouse model, we found that attenuated TGFβ signaling in epithelial cells correlated with decreased CXCL1, CXCL5, MCP-1, IL-6 and increased VEGF expression. Therefore, we first conducted analysis of correlation between expression of the *TGFBR2* gene and these genes. We found that expression of CXCL1, but not CXCL5, decreased significantly in patients with low *TGFBR2* expression (Figure 5A, B), but when we analyzed subtypes of tumor we discovered significant differences only in HER2+, PR + and ER + patients. There was no change in PR-negative (PR-) and ER- patients. MCP-1 and IL-6 expression decreased in all patients with low levels of *TGFBR2* expression, with no differences in subtypes of breast tumor (Figure 5C). By checking the association between *VEGFA* and *TGFBR2* expression using the TCGA BRCA dataset, we detected an opposite effect to that seen with CXCL1, MCP-1 and IL-6. This inference is consistent with our findings in a mouse model. VEGF expression increased in patients with all types of tumors with low *TGFBR2* expression (Figure 5C). We further validated these analyses using PAM50 enrichment of HER2 patients from the aforementioned six human breast cancer GEO microarray datasets. The majority of the results are consistent with our previous findings from the TCGA data (Additional file 1: Figure S6). In all six datasets, attenuated TGFβ signaling was correlated with low CXCL1 expression among HER2-enriched patients. Attenuated TGFβ signaling was also significantly correlated with low VEGF expression among all patients and HER2-enriched patients in GSE10886; however, this conclusion was not validated in other datasets.

In our current study using the GEM tumor model, we indicated that metastasis is a major phenotype in mice with abrogated TGFβ signaling in the tumor epithelium. Next, we examined the association of the *VEGF* gene with tumor subtypes and DMFS and noted a significant association between high *VEGFA* expression and worse DMFS in HER2+ patients (Figure 5D). Nonetheless, there was not a statistically significant difference in patients with ER+, PR+, or other subtypes of breast cancer.

This research finding indicates that attenuated TGFβ signaling correlates with decreased expression of CXCL1 and increased VEGF expression and is associated with worse survival rates in HER2+ patients. The aforementioned results were confirmed on the Neu-induced mouse tumor and human breast cancer models.

## Discussion

Important roles of TGFβ and HER2 signaling in tumor initiation and progression have been established in a large number of studies. To examine the role of TGFβ signaling in HER2+ breast cancer, we used MMTV-Neu mice with DNIIR. In our studies 202Mul mice with overexpression of wild-type ErbB2 and NK1Mul mice with mutant activated ErbB2 were utilized. In our experiment, we did not incorporate any specific pathways as was executed in the Siegel *at al*. publication [16]. In our analysis of the TCGA database (Additional file 1: Figure S7) we observed that in fewer than 5% of patients ErbB2 mutated and in about 15% of patients the ErbB2 receptor was amplified. This result was supported by recently published work by Bose *et al*. [34], in which researchers observed ErbB2 mutation in only a small percentage of HER2+ patients. Based on this fact we believe that our GEM tumor model is highly appropriate for investigation of the HER2+ type of breast cancer with attenuated TGFβ signaling.

In our GEM model the major differences between mice with intact and disrupted TGFβ signaling were increased tumor latency in parallel with the increased number of lung metastases (Figure 1). Changes in metastasis were different when compared with mouse models where ErbB2 was mutated by activated Grb2 or Shc signaling pathways [16]. The increased tumor latency was also opposite to MMTV-PyMT/TGFβRII-KO mice with deletion of *Tgfbr2* in the mammary epithelium [5], as previously studied in our laboratory. However, the increase in lung metastases in the MMTV-Neu/DNIIR mouse model was similar to MMTV-PyMT/TGFβRII-KO mice. This indicates that any manipulation to diminish TGFβ signaling in GEM models will lead to increased metastasis regardless of tumor-driving oncogenic transformations.

Increased tumor latency in MMTV-Neu/DNIIR mice (202Mul/DN, NK1Mul/DN) versus control mice (202Mul, NK1Mul) could be first due to cell-cycle dysregulation, where TGFβ signaling plays an important role, and second, due to dysregulation of chemokine expression. Feng *et al*. demonstrated that the immune cells provide a source of tropic support to transformed epithelial cells, just as they do to normal epithelium during wound healing, and play a primary role in tumor initiation [35]. We found that mice with DNIIR expression had downregulated levels of CXCL1 and CXCL5 (Figure 3) and as a result, fewer CD11b^+^Gr1^+^ cells in tumor tissue (Additional file 1: Figure S3).

Based on our current study and Siegel *et al*. [19] we can make a basic conclusion that attenuated TGFβ signaling in HER2+ tumor models, with active Shc and Grb2 pathways, decreases the probability of lung metastasis development. With intact ErbB2, attenuated TGFβ signaling has the opposite effect in spite of the fact that tumor latency increases.

Tumor tissues in mice with DNIIR had increased vasculogenesis, increased vessel size, leakiness, and decreased number of pericytes (Figure 2). Clinical data showed that a low number of pericytes correlated with poor patient prognosis [36],[37]. Simultaneously, disruption of pericytes also enhanced metastasis [38]. In parallel with a decreased number of pericytes, we discovered that the size of vessels in tumor tissue was larger in mice with DNIIR. We hypothesized that there were probably two different mechanisms involved in increased vasculogenesis in DNIIR mice; and increased angiogenesis in parallel with the disruption of vessel support by pericytes. It is likely that an increase in vessel leakage leads to an increased number of metastases [38].

In our previously published articles, we indicated that deletion of *Tgfbr2* leads to an increase in chemokine expression in mammary and pancreatic epithelium [7],[39]. Researchers have also linked deletion of *Tgfbr2* to an increase in mammary fibroblasts [40]. In the mammary tumorigenesis studies, the major differences were found in CXCL1 and CXCL5 expression, which play an important role in the migration of neutrophils and myeloid-derived suppressor cells (CD11b^+^Gr1^+^).

An increased number of myeloid cells in MMTV-PyMT mice could be a basic mechanism driving decreased tumor latency and increased number of lung metastases. In our current study with attenuated TGFβ signaling in MMTV-c-Neu mice, we discovered an opposite effect in which levels of CXCL1/CXCL5 as well as CCL2 (MCP-1) decreased (Figure 3) in parallel with an increased level of VEGF. Comparison analysis of chemokine and VEGF secretions in MMTV-PyMT cells with DNIIR (Additional file 1: Figure S3) showed the same effect. There was an opposite result when TGFβRII was deleted. Our laboratory published a study [41], which showed that DNIIR system could not completely inhibit the non-canonical TGFβ pathway versus the canonical (SMAD-dependent). This information correlates with our data on increased pAKT (Figure 4, Additional file 1: Figure S5) in MMTV-Neu/DNIIR cells, which is downstream of the non-canonical TGFβ pathway. We propose that it is a significant mechanism in the differential regulation of chemokines and VEGF secretion. We can conclude that the MMTV-Neu/DNIIR GEM tumor model is a model of spontaneous mammary carcinogenesis with a diminished canonical TGFβ pathway (SMAD-dependent) with the still-active non-canonical TGFβ pathway. There is also a significant amount of data to support a dose-response mediating TGFβ-induced phenotypic changes [42],[43]. Thus, it is likely that the observed gene expression changes are the result of altered TGFβ response due to the specific amount of TGFβ induced in cells, which express the DNIIR. Such observations could not be made with conditional knockout of *Tgfbr2* as TGFβ signaling is completely abrogated. Other authors also found increased levels of pAKT when combining activated TGFβ signaling (ALK5^T204D^, TGFβ1^S223/225^) with Neu-induced tumorigenesis [15],[17]. It can be explained by overactivation of both the canonical and non-canonical TGFβ pathways. An increased level of VEGF can explain increased vasculogenesis and vessel leakage in MMV/c-Neu DNIIR mice and could likely be involved in the observed increase in lung metastases.

Previously, we found that deletion of *Tgfbr2* leads to an increase in the number of CD11b^+^Gr1^+^ cells in MMTV-PyMT mice. In Neu-induced carcinogenesis, we did not observe many differences in immune components in mice with DNIIR versus control mice. The number of CD11b^+^Gr1^+^ cells decreased in tumor tissue from MMTV-c-Neu DNIIR mice that could result from downregulation of chemokines. Simultaneously the number of T cells decreased in parallel with the number of CD11b^+^Gr1^+^ cells in the MMTV-c-neu DNIIR tumors. Usually, increased numbers of CD11b^+^Gr1^+^ cells correlate with suppression of T cell proliferation [44], but in our model we did not observe these changes to take place. Therefore, we suggest that decreased number of T cells could be due to the increased VEGF secretion in DNIIR mice. It has been shown that VEGF strongly inhibits T-cell development via VEGFR2 [32]. Also, VEGF receptors are capable of inhibiting dendritic cell function and, thus, we hypothesized that anti-tumor immune response in mice with DNIIR diminished as a result of higher levels of VEGF.

Based on these findings, we conclude that human HER2+ breast cancer associated with decreased TGFβ signaling would also correlate with deceased expression of CXCL1/5 chemokines and increased VEGF. We observed that decreased *TGFBR2* expression in human breast cancer patients correlated with decreased CXCL1, but not with CXCL5 in HER2+, PR + and ER + tumors. MCP-1 and IL6 decreased and VEGF level increased in all types of breast tumors with low *TGFBR2* expression. To our surprise, increased VEGF expression in human breast cancer patients correlated with reduced DMFS only in HER2+ patients. The same outcomes were observed in our mouse studies.

## Conclusion

Our results demonstrated that attenuation of TGFβ signaling in HER2+ mammary epithelium delays tumor initiation, but promotes lung metastasis. The mechanism behind this phenomenon appears to be due to the increased VEGF secretion in parallel with deceased CXCL1 and CXCL5 secretion. The result of increased VEGF is increased tumor angiogenesis and vessel leakage that leads to an increase in lung metastasis. Also, increased VEGF secretion inhibits T cell proliferation and potentially inhibits anti-tumor immune response. Our studies provide insights into a novel mechanism by which epithelial TGFβ signaling modulates the tumor microenvironment and is involved in lung metastasis in HER2+ breast cancer patients. The effects of pharmacological targeting of the TGFβ pathway *in vivo* during tumor progression remain controversial because of the dual role TGFβ plays in tumor development. The targeting of TGFβ signaling should be considered as a viable option, but because VEGF has a pro-tumorigenic effect on HER2+ tumors, the targeting of this protein could be considered only when it is associated with attenuated TGFβ signaling.

## Additional file

## Electronic supplementary material

Additional file 1: Figure S1.: CD34 immunohistochemistry analysis of mouse lungs. **Figure S2.** **(A)** Fluorescence-activated cell sorting (FACS) dot plots of pericyte analysis. FACS analysis of pericytes (CD140b + CD140a-) in single-cell suspension of tumor tissue. Cells were gated as alive (4',6-diamidino-2-phenylindole (DAPI)-) and gated as non-immune, non-epithelial (CD45-CD326-) as shown on the right side. **(B)** Distribution of tumor vessels within specified size ranges. Number of vessels detected on area equals 50,000 um2. **Figure S3.** ELISA data. **Figure S4.** Number of T cells and myeloid cells in spleen and tumor tissue. **Figure S5.** Western blot analysis of cultured cells. **Figure S6.** **(A)** The summary table of the additional datasets we have been observed; 1, data available; 0, data not available. To replicate TCGA data we used the GSE10886 dataset, but it contains information only from 220 patients and only 11 are human epidermal growth factor receptor 2 (HER2)+. **(B)** The replication of plot 5A-C in the independent dataset GSE10886. Our findings in the manuscript are well-replicated in this dataset; however, as the new dataset sample size is small (in particular there are numerous missing values for estrogen receptor (ER), progesterone receptor (PR) and HER2), some of the *P*-values are not significant. **Figure S7.** Analysis (Cbioportal.org) of TCGA breast cancer molecular database for ErbB2 status. Bar graph depicts percentage of patients with either amplified or mutated ErbB2 of the total HER2+ patients. *Comprehensive molecular portraits of human breast tumors. Nature, 2012. 490(7418): p. 61-70* (PDF 656 kb); (http://breast-cancer-research.com/content/supplementary/s13058-014-0425-7-s1.pdf). (PDF 657 KB)

Below are the links to the authors’ original submitted files for images.

Authors’ original file for figure 1

Authors’ original file for figure 2

Authors’ original file for figure 3

Authors’ original file for figure 4

Authors’ original file for figure 5

## Abbreviations

BrdU: 5-bromo-2’-deoxyuridine
BSA: bovine serum albumin
CP: collagen plugs
DAPI: 4',6-diamidino-2-phenylindole
DMEM: Dulbecco's modified Eagle's medium
DMFS: distant metastasis-free survival
DNIIR: (or DN): dominant-negative TGFβ receptor II
ELISA: enzyme-linked immunosorbent assay
ER: estrogen receptor
FACS: fluorescence-activated cell sorting
FITC: fluorescein isothiocyanate
H&E: hematoxylin and eosin
IF: immunofluorescence
IHC: immunohistochemistry
IL: interleukin
MCP: monocyte chemotactic protein
MMTV: mouse mammary tumor virus
Neu: (also known as ErbB2) human epidermal growth factor receptor 2 (HER2)
PR: progesterone receptor
PyMT: Polyoma middle T antigen
TGFβ: transforming growth factor beta
TβRI: transforming growth factor β receptor I
TβRII: transforming growth factor β receptor II TIMP: tissue inhibitor of metalloproteinase
VEGF: vascular endothelial growth factor
