# Supplementary figures and images for: Attenuated transforming growth factor beta signaling promotes metastasis in a model of HER2 mammary carcinogenesis
Source: Breast Cancer Res. 2014 Oct 4;16:425. doi: 10.1186/s13058-014-0425-7 (PMC4303109; doi:10.1186/s13058-014-0425-7)

202Mul

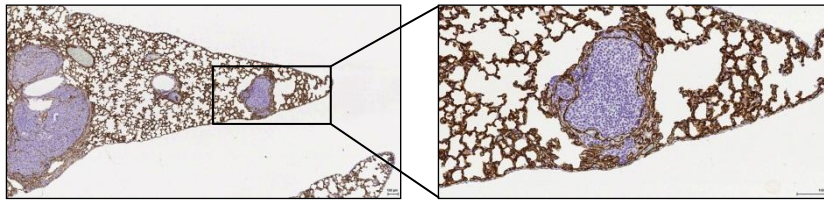

202Mul/DN

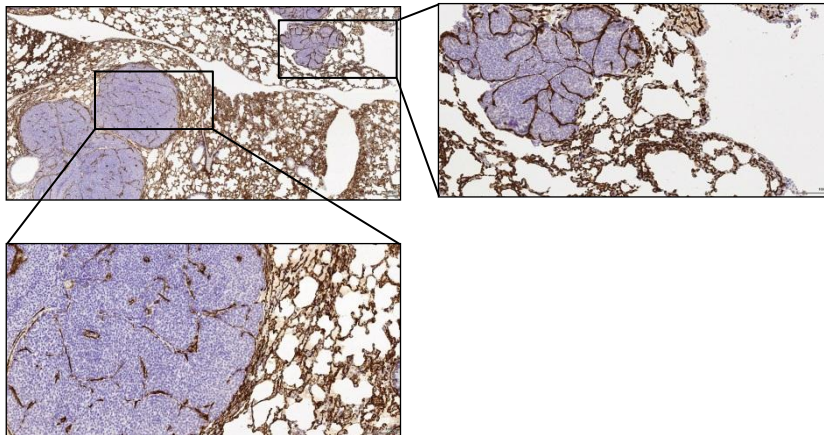

A

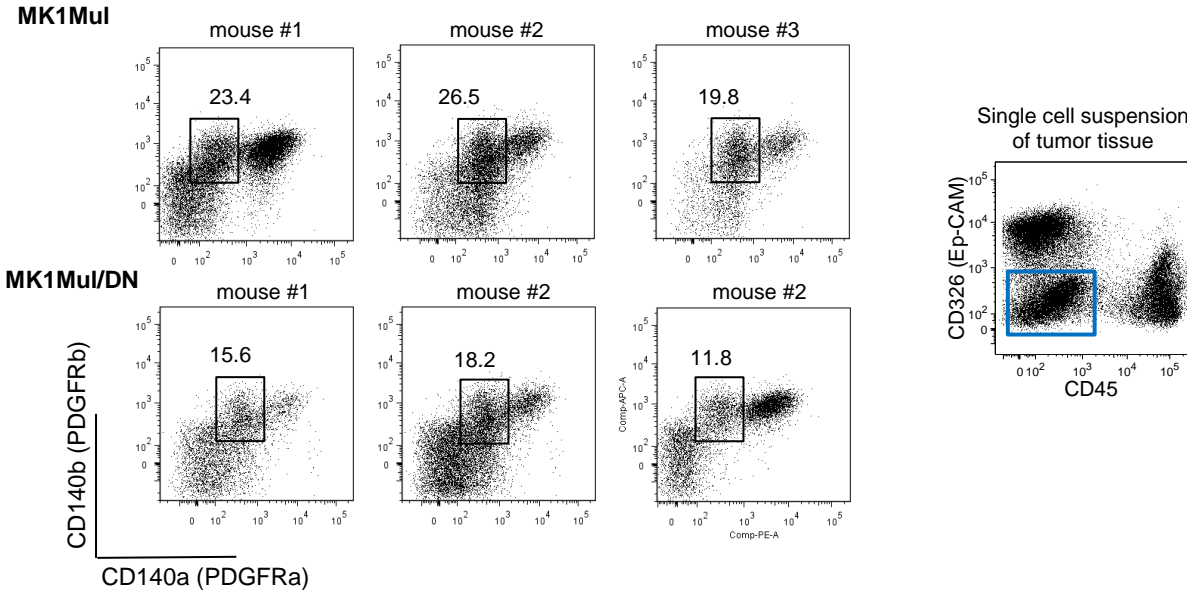

B

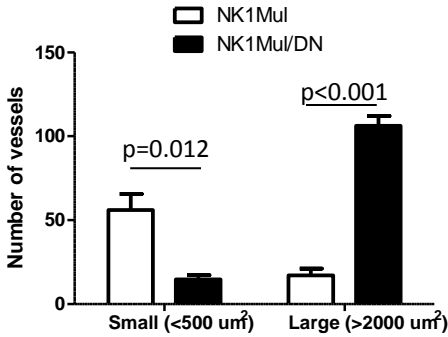

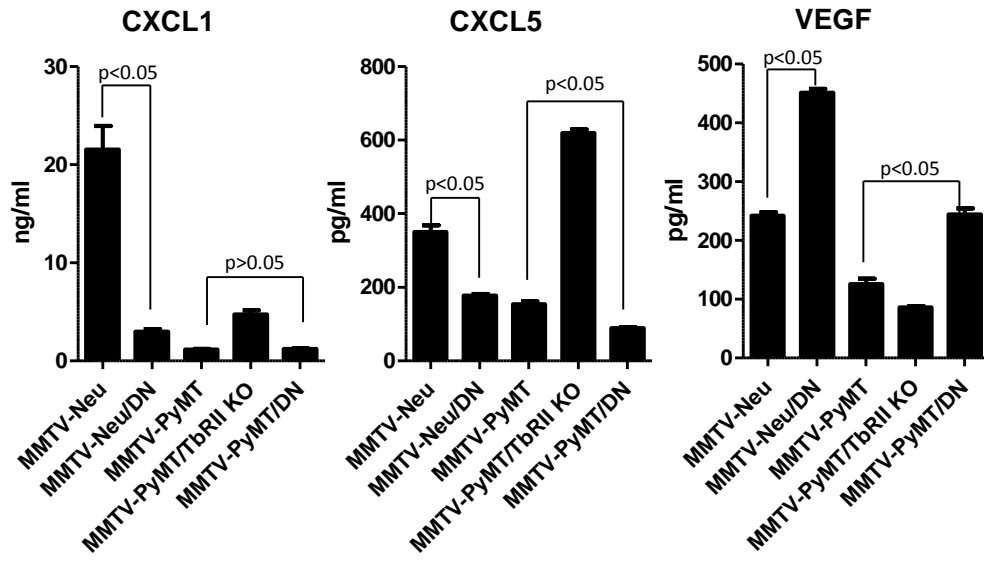

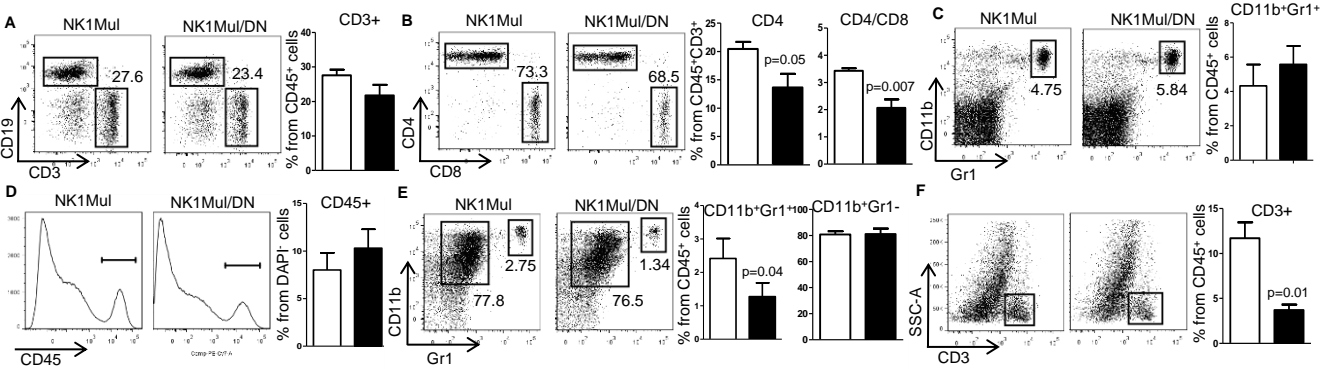

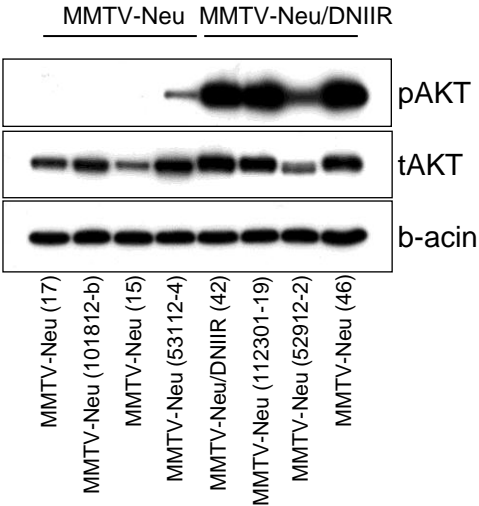

A

|          | HER2 | DMFS |
|----------|------|------|
| NKI-295  | 0    | 1    |
| GSE10886 | 1    | 0    |
| GSE4922  | 0    | 0    |
| GSE6532  | 0    | 1    |
| GSE2990  | 0    | 1    |
| GSE2845  | 0    | 0    |
| GSE12093 | 0    | 0    |

B

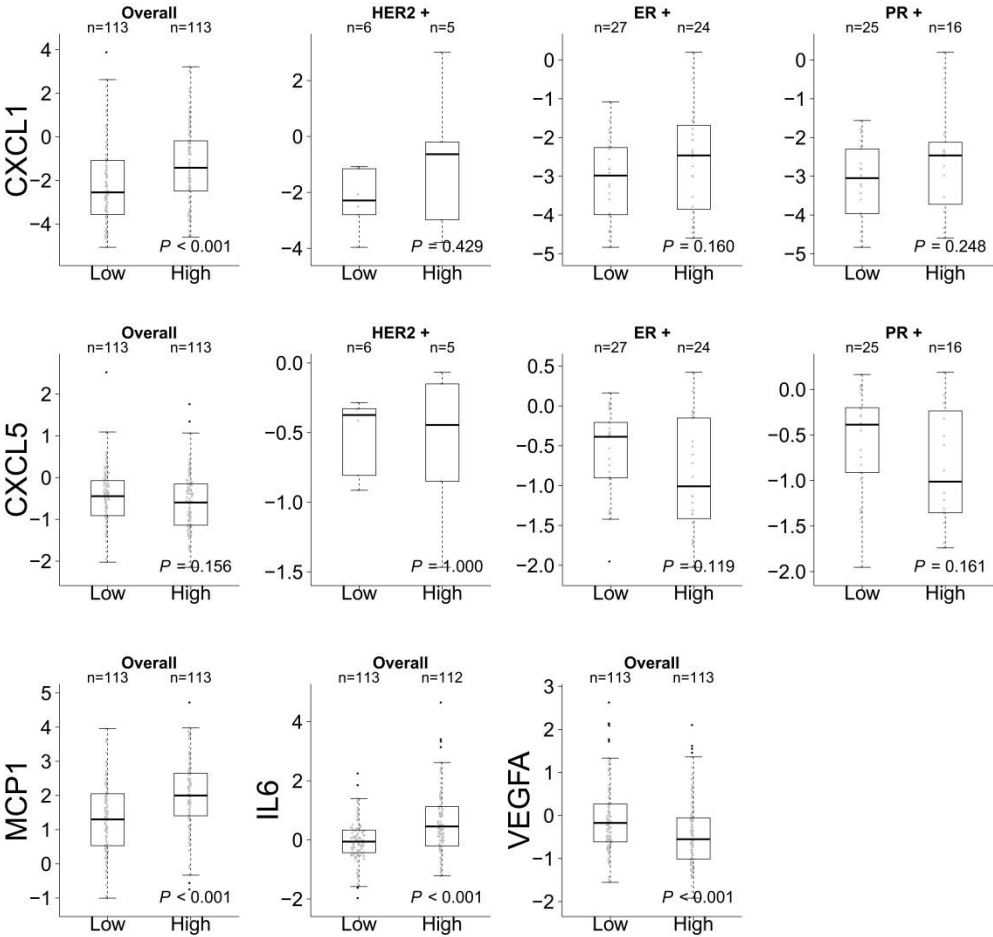

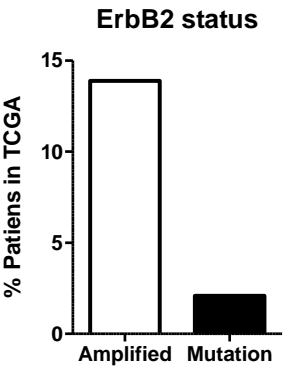

Supplement: Supplementary file 1 — Additional file 1: Figure S1.: CD34 immunohistochemistry analysis of mouse lungs. Figure S2. (A) Fluorescence-activated cell sorting (FACS) dot plots of pericyte analysis. FACS analysis of pericytes (CD140b + CD140a-) in single-cell suspension of tumor tissue. Cells were gated as alive (4',6-diamidino-2-phenylindole (DAPI)-) and gated as non-immune, non-epithelial (CD45-CD326-) as shown on the right side. (B) Distribution of tumor vessels within specified size ranges. Number of vessels detected on area equals 50,000 um2. Figure S3. ELISA data. Figure S4. Number of T cells and myeloid cells in spleen and tumor tissue. Figure S5. Western blot analysis of cultured cells. Figure S6. (A) The summary table of the additional datasets we have been observed; 1, data available; 0, data not available. To replicate TCGA data we used the GSE10886 dataset, but it contains information only from 220 patients and only 11 are human epidermal growth factor receptor 2 (HER2)+. (B) The replication of plot 5A-C in the independent dataset GSE10886. Our findings in the manuscript are well-replicated in this dataset; however, as the new dataset sample size is small (in particular there are numerous missing values for estrogen receptor (ER), progesterone receptor (PR) and HER2), some of the P-values are not significant. Figure S7. Analysis (Cbioportal.org) of TCGA breast cancer molecular database for ErbB2 status. Bar graph depicts percentage of patients with either amplified or mutated ErbB2 of the total HER2+ patients. Comprehensive molecular portraits of human breast tumors. Nature, 2012. 490(7418): p. 61-70 (PDF 656 kb); (http://breast-cancer-research.com/content/supplementary/s13058-014-0425-7-s1.pdf). (PDF 657 KB) [file 13058_2014_425_MOESM1_ESM.pdf]

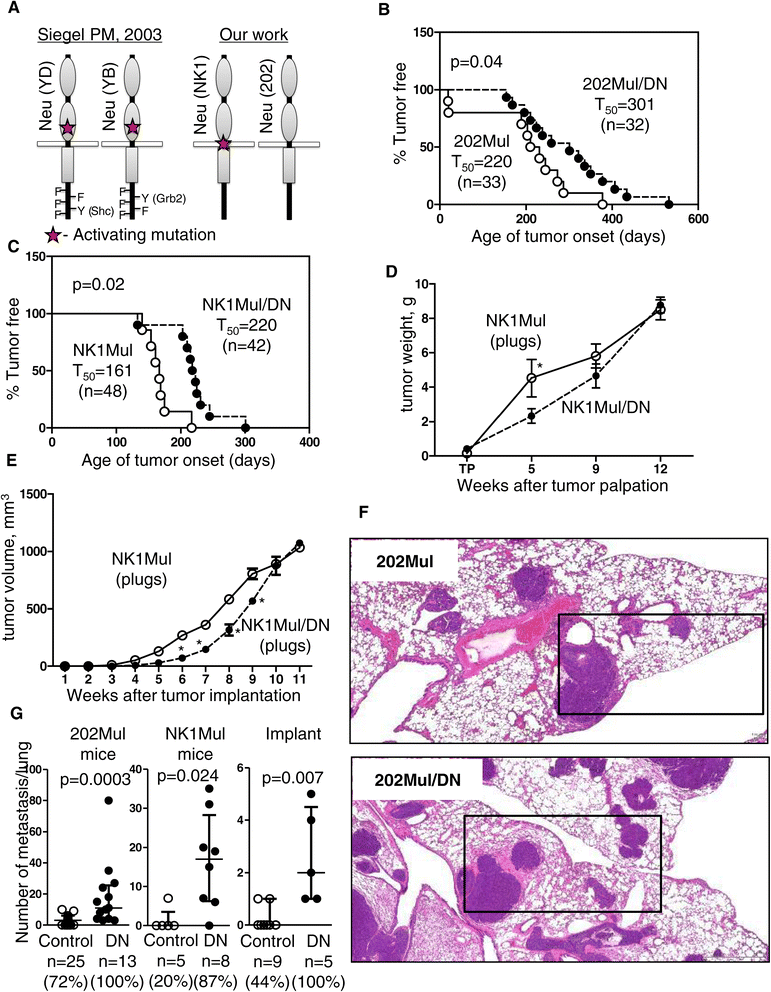

Supplement: Supplementary file 2 — Authors’ original file for figure 1 [file 13058_2014_425_MOESM2_ESM.gif]

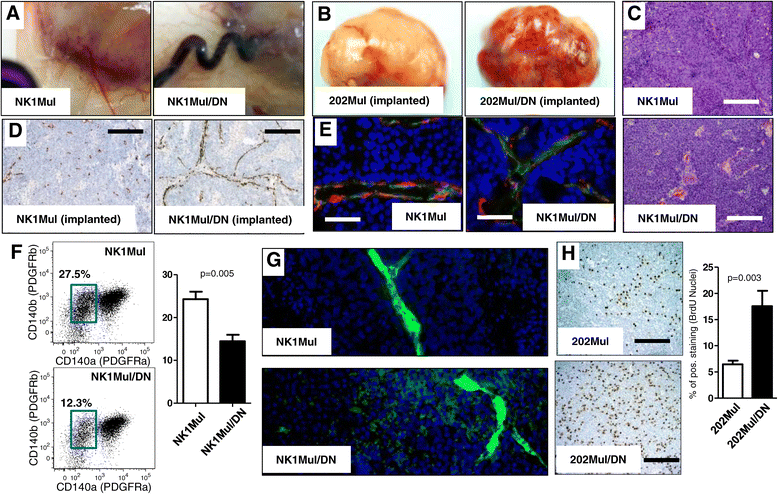

Supplement: Supplementary file 3 — Authors’ original file for figure 2 [file 13058_2014_425_MOESM3_ESM.gif]

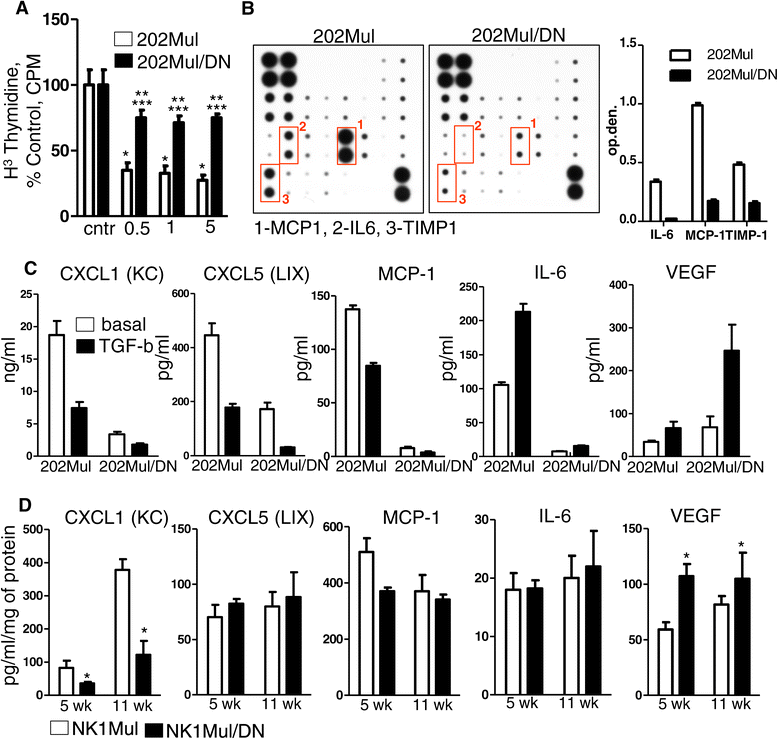

Supplement: Supplementary file 4 — Authors’ original file for figure 3 [file 13058_2014_425_MOESM4_ESM.gif]

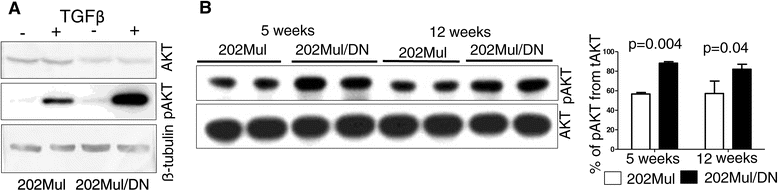

Supplement: Supplementary file 5 — Authors’ original file for figure 4 [file 13058_2014_425_MOESM5_ESM.gif]

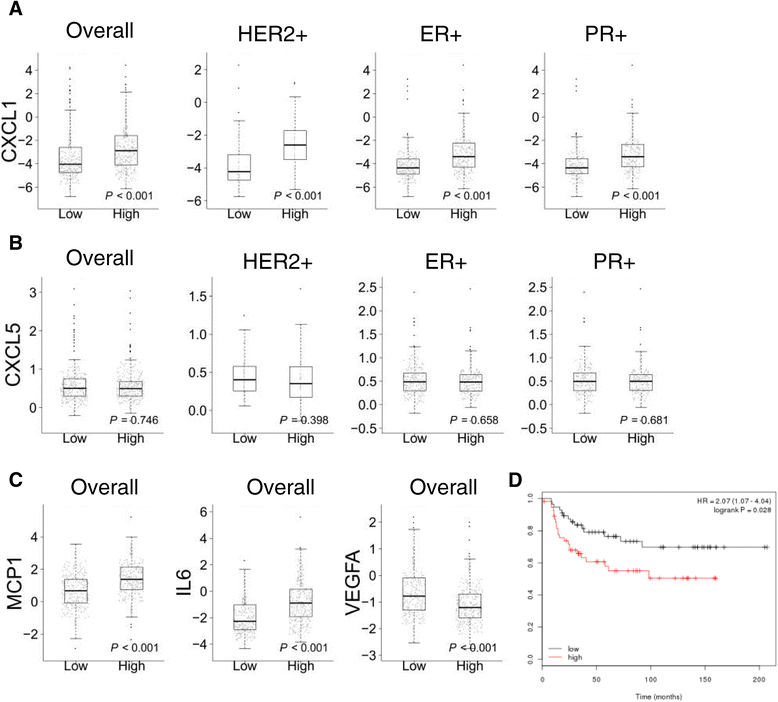

Supplement: Supplementary file 6 — Authors’ original file for figure 5 [file 13058_2014_425_MOESM6_ESM.gif]
